# Upholding the right to health of forcibly displaced children in Europe

**DOI:** 10.1016/j.puhip.2025.100641

**Published:** 2025-07-07

**Authors:** Amy Stevens, Zeliha Öcek, Sergey Sargsyan, Michelle Black

**Affiliations:** a Bevan Healthcare, UK; b Institute for Medical Information Processing, Biometry, and Epidemiology (IBE), Chair for Public Health and Health Services Research, Faculty of Medicine, LMU Munich, Munich, Germany; c Arabkir Medical Centre – Institute of Child and Adolescent Health, Yerevan State Medical University, Armenia; d Public Health Policy and Systems, University of Liverpool, UK

**Keywords:** Child, Migrant, Asylum, Refugee, Health, Policy

## Abstract

In 2023 Europe hosted an estimated 9 million children who had been forcibly displaced from their homes because of conflict, persecution, violence, natural or environmental disasters, climate crisis, human trafficking and extreme poverty. Their experiences pre, during and post migration impact their health, wellbeing and development. Countries across the European Region have a moral and legal duty to uphold the right to health of all children living within their borders, irrespective of immigration status. However, many countries are falling short of delivering on these obligations. The rise in populist radical right politics and anti-immigrant sentiment across the Region has led to an increase in potentially health-harming immigration policies and practices. Challenges to meeting the health needs of displaced children include underfunded health systems, limited specialist services, health and care workforce shortages, and lack of data to inform evidence-based policy and practice. Displaced children are often subjected to restrictions on service entitlements; systemic racism, xenophobia and discrimination in health systems; and language, cultural, social, financial, and administrative barriers to care. Cross-country collaboration is required to address the drivers of forced migration; increase availability of safe and legal routes for refugees; and ensure health systems across the Region have the data, resource and capacity required to respond to the needs of displaced children. Essential policies supporting a child's right to health include: provision of child and family-centred community alternatives to refugee camps and immigration detention; provision of healthcare and education entitlements equitable to children of the host nation; protection of children from violence and exploitation; and delivery of quality and inclusive trauma-informed healthcare that accounts for language needs, cultural diversity and safeguarding risks. With political commitment and coordinated efforts, ensuring the right to health for displaced children is achievable and should be prioritised.

## 1 Introduction

Global geopolitical, economic and climate crises are having a catastrophic impact on children. In 2023 Europe hosted an estimated 9 million children who had been forcibly displaced because of conflict, persecution, violence, natural or environmental disasters, climate crisis impacts, abuse, exploitation, human trafficking, threat of child marriage and extreme poverty [[1], [2], [3]].A figure which has doubled in the four years from 2019 to 2023 [4]. Countries have a moral and legal duty to act. All countries in the World Health Organization (WHO) European Region (53 states across Europe and Central Asia as listed in Panel 1) are signatories to the United Nations Convention on the Rights of the Child (UNCRC) [5]. They have a responsibility to protect the rights of all children, including ‘*the right of the child to the enjoyment of the highest attainable standard of health and to facilities for the treatment of illness and rehabilitation of health’,* irrespective of their nationality, immigration status or statelessness [5]. Successfully upholding the rights of children who have been forcibly displaced requires cross country collaboration, national government commitment, and inclusive, accessible and adequately resourced public services. Taking a collaborative Regional approach to the challenge presents an opportunity to harness the power of WHO and UNICEF, as international agencies, to advocate for children's rights and leverage cross country action. Leaders and policy-makers need to understand the current situation facing displaced children across the Region and appreciate how political choices can increase their vulnerability. Healthcare service providers should be aware of how the experiences of displaced children may impact their health and what action is required to ensure delivery of equitable and quality services to meet these needs. To this end, we present an overview of the number and demographics of children forcibly displaced and the migration journeys they make across international borders in the WHO European Region. We discuss the challenges presented by health harming immigration policy together with opportunities for action to uphold their rights to health and facilities.

## 2 The risks facing children who have been forcibly displaced

Children who have been forcibly displaced include refugees, asylum seekers, undocumented migrants seeking sanctuary and internally displaced children within countries. While their needs often overlap there are important differences between these groups in terms of their legal rights and public service entitlements; definitions are given in Panel 1.

**Table:** 

Panel 1: Key definitions
Child
Every human being below the age of eighteen years unless under the law applicable to the child, majority is attained earlier [5].
Migrant
An umbrella term, not defined under international law, reflecting the common lay understanding of a person who moves away from his or her place of usual residence, whether within a country or across an international border, temporarily or permanently, and for a variety of reasons [6].
Asylum-seeker
An individual who is seeking international protection. In countries with individualised procedures, an asylum seeker is someone whose claim has not yet been finally decided on by the country in which he or she has submitted it [6].
Refugee
A person who, owing to a well-founded fear of persecution for reasons of race, religion, nationality, membership of a particular social group or political opinion, is outside the country of his nationality and is unable or, owing to such fear, is unwilling to avail himself of the protection of that country; or who, not having a nationality and being outside the country of his former habitual residence as a result of such events, is unable or, owing to such fear, is unwilling to return to it [6].
Undocumented migrant
A non-national who enters or stays in a country without the appropriate documentation. This may include trafficked children and children born to undocumented migrant parents [6].
Unaccompanied minors
Children who have been separated from both parents and other relatives and are not being cared for by an adult who, by law or custom, is responsible for doing so [6].
Internally displaced person
Persons or groups of persons who have been forced or obliged to flee or to leave their homes or places of habitual residence, in particular as a result of or in order to avoid the effects of armed conflict, situations of generalized violence, violations of human rights or natural or human-made disasters, and who have not crossed an internationally recognized State border [6].
Stateless person
A person who is not considered as a national by any State under the operation of its law [6].
Trafficked child
Child who has been recruited, transported, transferred, harboured or received for the purpose of exploitation [6].
World Health Organization (WHO) European Region
The WHO European Region comprises of 53 states: Albania, Andorra, Armenia, Austria, Azerbaijan, Belarus, Belgium, Bosnia and Herzegovina, Bulgaria, Croatia, Cyprus, Czech Republic, Denmark, Estonia, Finland, France, Georgia, Germany, Greece, Hungary, Iceland, Ireland, Israel, Italy, Kazakhstan, Kyrgyzstan, Latvia, Lithuania, Luxembourg, Malta, Moldova, Monaco, Montenegro, Netherlands, North Macedonia, Norway, Poland, Portugal, Romania, Russia, San Marino, Serbia, Slovakia, Slovenia, Spain, Sweden, Switzerland, Tajikistan, Türkiye, Turkmenistan, Ukraine, United Kingdom, Uzbekistan. Where this paper cites the ‘Region’ it is referring to the WHO European Region.
Non-refoulement principle
Under international human rights law, the principle of non-refoulement guarantees that no one, irrespective of migration status, should be returned to a country where they would face torture, cruel, inhuman or degrading treatment or punishment and other irreparable harm [6].

Children who have been forcibly displaced are exposed to multiple health harming risks and violations of their rights as a result of their experiences pre, during and post migration, as shown in Table 1 [7]. Associated adverse outcomes include communicable diseases, malnutrition, injuries, psychological trauma, and delayed/inadequate management of health needs, all of which can have potentially life-long impacts on a child's physical and mental health, wellbeing, growth, development, and risk of disability.

**Table 1:** Health risks pre, during and post migration (Adapted from Stevens (2020) [7]).

Premigration	Migration	Post migration
• War and conflict	• Long physically demanding and dangerous journeys	• Hostile anti-immigration policies
• Persecution	• Physical and sexual violence	• Immigration detention
• Environmental hazards	• Overcrowded refugee camps	• Long and complex legal immigration process
• Limited and disrupted healthcare	• Exploitation by traffickers and people smugglers	• Restricted rights and public service entitlements
• Poverty	• Modern slavery	• New language
• Famine and undernutrition	• Limited access to adequate food, sanitation, shelter and healthcare	• New culture
• Loss of shelter	• Interrupted routine childhood vaccination	• Loneliness and isolation
• Endemic disease	• Endemic disease	• Loss of identity and status
• Physical and sexual violence	• Family separation	• Parentlessness and absence of family
• Torture	• Violent pushbacks at borders	• Lack of community support
• Witnessing violence/torture/death	• Missed education	• Poverty
• Loss and bereavement	• Poor maternal health	• Poor housing
• Forced recruitment to armed groups	• Poor caregiver health	• Healthcare access barriers
• Poor maternal health impacting pregnancy and birth outcomes and ability to breastfeed		• Delay in accessing education
• Poor caregiver health		• Racism, xenophobia and discrimination
		• Risk of exploitation and trafficking
		• Child labour
		• Poor maternal health
		• Poor caregiver health

## 3 Numbers and demographics of forcibly displaced children in Europe

**Fig. 1:**
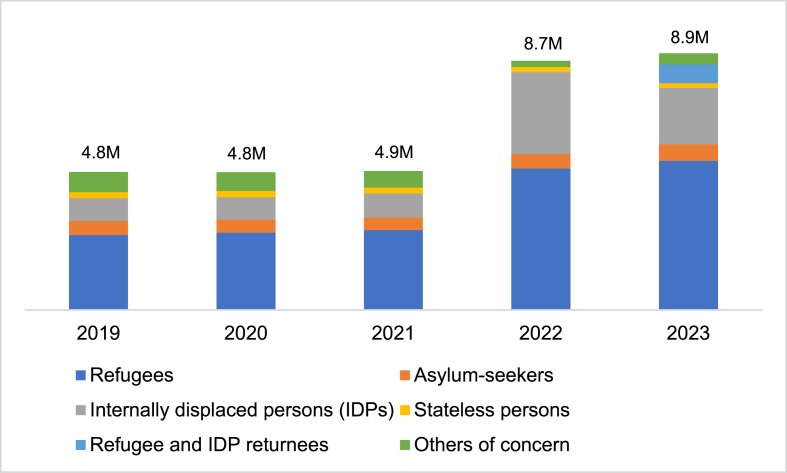
Displacement trend in Europe 2019–2023 with estimated displaced child population totals [3].

The numbers and nationalities of children forcibly displaced across international borders reflects conflicts and global insecurities happening at any given time. Precise data for the WHO European Region is limited as many children arrive by irregular routes. Where population data does exist, it is often incomplete and limited by lack of age disaggregation and immigration status. Published figures are likely underestimates of the true number of children seeking sanctuary in the Region but trends and available demographic data can give an indication of the situation in Europe (Fig. 1). In 2023 there were 254,900 first time child asylum-applications in European Union (EU) countries, the highest number since the 2016 “migration crisis” [8]. Forty-four percent originated from Asian countries, 20.2 % from European (non-EU) countries, 19.8 % from African countries and 14.4 % from American countries; the most represented citizenships were Syrians and Afghans [9]. 58.6 % were male, 72.9 % were aged under 14 years and 17 % were unaccompanied minors [8].

Bar chart data source: UNHCR total population data; estimates of displaced child population totals calculated using UNHCR estimation that 40 % of globally displaced persons are children. The UNHCR Refugee Data Finder does not contain data for eight of the WHO European Region States (Andorra, Israel, Kazakhstan, Kyrgyzstan, San Merino, Tajikistan, Turkmenistan and Uzbekistan).

The distribution of displaced children across the WHO European Region is variable and influenced by geographical, economic and political factors, cultural proximity (e.g. common language, previous colonial ties) and social networks. Within the EU, Germany (40.6 %), France (15.1 %) and Spain (12 %) were the top three destination countries for first time asylum applicant children in 2023 [8]. While there is country level data on the number of child asylum applications, data on the number of displaced children in transit to their destination countries is less readily available. Numbers are likely highest in countries bordering conflict areas and along common migration routes into and through Europe. Türkiye hosts the largest child refugee population, not only within the Region, but globally [10]. In 2023 Türkiye was home to 1.7 million child refugees, most having fled conflict in the Syrian Arabic Republic [10]. More than 2 million children have fled Ukraine following the 2022 Russian Federation invasion and ongoing war [11]. Many Ukrainian refugees have been granted temporary protection across Europe, with the highest numbers hosted in Germany, Poland and the Czech Republic [12]. Hundreds of thousands of Ukrainian children have reportedly been forcibly displaced or deported into the Russian Federation where there are concerns about their legal status, rights and access to services [12]. In 2024 36,000 children were seeking refuge in Armenia due to hostilities in the South Caucasus region [13].

## 4 Migration journeys to and through Europe

Safe and legal (regular) routes by which children who have been forcibly displaced can enter European countries, for example resettlement and family reunion pathways, are inaccessible to most children seeking sanctuary in Europe. Instead, many are forced to enter the Region by irregular routes and apply for asylum on arrival to their destination country. Irregular migration journeys to and across Europe are often long, difficult and dangerous. The Central Mediterranean Sea has become one of the most dangerous routes travelled by children with 1500 children having died or gone missing while attempting this sea crossing between 2018 and 2023 [14]. Perilous sea crossings and challenging terrain are not the only risks to children during migration. They often face food insecurity, lack of shelter, overcrowded refugee camps, exposure to communicable diseases, and limited access to water, sanitation and healthcare [7]. Their journeys may expose them to physical and sexual violence, abuse, exploitation by traffickers and people smugglers, forced labour, and organ harvesting. Risks are heightened for unaccompanied and separated children who may have been orphaned, become separated from relatives during their migration journey, or have been forced to leave home without their families [15]. Between 2021 and 2023, over 50,000 unaccompanied refugee children have been registered as missing in EU countries, with concerns that many may have been exploited and abused for sexual or labour purposes [16].

Some child migrants may be trafficked into the Region against their will or may become victims of human trafficking after arrival in a European country. Where the victim's age group is known, 1500 of the 10,000 registered victims of human trafficking in the EU in 2022 were children, 75 % of whom were girls; an estimated 75 % of registered victims originated from another country [17].

## 5 Upholding the right of the child to the enjoyment of the highest attainable standard of health

### 5.1 Addressing the potential health harming impact of host countries politics, policies and practices

Despite committing to upholding the rights of children, the national politics and policies of many European Region countries have potential to exacerbate the health needs and vulnerabilities of displaced children, as a direct consequence of policy, e.g. restricted access to healthcare, or indirectly, through social and economic pathways [18]. The negative political rhetoric around migration increases racism, xenophobia and discrimination experienced by child migrants at interpersonal, institutional and structural levels, contributing to the health inequities they experience [19].

Over recent years there has been an increase in populist radical right politics and anti-immigrant sentiment across the Region, perhaps emerging from political parties' failures to effectively manage migration and address increasing socioeconomic inequality. Forcibly displaced people have become scapegoats for these failures. This has led to a narrative of migration as a bad thing despite history demonstrating the opposite. Well managed migration drives development and prosperity. Lack of planning and cross-country collaboration has led to pressure on services and public discontent which in turn allows a political opportunity to limit migrants' entitlements and access to: protection; healthcare; public services; financial support; housing; education; and employment opportunities. Additionally, a background of widening socioeconomic inequalities against the increase in immigration to the Region has heightened population concerns about labour market competition and compositional amenities, and decreased support for redistribution [20]. The result of this failure is a defensive approach to managing migration and opportunities for radical right parties to promote nativism to attract the votes of those who have been ‘left behind’ with a promise to lift their status with a non-economic source of prestige [21]. Children are innocent bystanders and this negative narrative around migration has meant their circumstances are not viewed as a humanitarian issue.

Multiple countries have contravened the non-refoulment principle and breached obligations to provide humane treatment and dignified reception centres at their borders [22]. There has been an increase in illegal and discriminatory pushbacks of people seeking asylum by EU Member State authorities at external borders, including in the waters south of Malta, Ceuta and Melilla in Northern Morocco, the Türkiye–Greece frontier, and the land borders of Croatia, Hungary, and Poland [23]. There is evidence of violent and degrading treatment towards asylum-seekers, torture, theft, detention, collective expulsions, and bilateral agreements with third countries to quickly return persons back [[23], [24], [25]]. Such activities violate human rights and international laws. Consequences include injury, psychological trauma, and death [24,25]. The International Organization for Migration reported at least 252 people died during alleged forced expulsions by European authorities between 2021 and 2022 [26]. The reported percentage of pushbacks where children are involved varies from 12 % to 53 % [23,25]. The Border Violence Monitoring Network publishes testimonies of pushbacks involving children.

The recently adopted EU Migration and Asylum Pact has attracted criticism that its fast-tracking measures could: result in children being denied asylum and protection they are entitled to; increase access barriers to essential services; heighten children's risk of violence, abuse and human trafficking; and increase pushbacks and systematic detention of families and children at external borders for screening purposes [27].

The authors acknowledge that management of large-scale migration flows caused by crises pose complex challenges for WHO European Member States. However, Regional cooperation can play a vital role in reducing the adverse effects of migration while safeguarding its integrity. International efforts to address the divers of forced displacement and effectively support populations at risk is a necessary long-term approach. Cross-country planning that utilises data to inform policy could facilitate effective migration governance and public health planning, and help realise the positive socioeconomic outcomes that migration can generate [28]. Combined with cross-sector government approaches at country level, European Regional commitment can make a child rights centred approach to migration possible.

Table 2 presents examples of Regional child health-harming immigration policies and practices, alongside recommendations for alternative approaches that uphold child rights and protect children's health and wellbeing.

**Table 2:** Examples of child health-harming immigration policies and practices and recommended alternatives.

Policy area	Description of concerning policies and practices in WHO European Region countries	Health impact	Recommendations
Migration pathways	There is a shortage of accessible regular migration pathways (legal and policy frameworks that enable people to move to, enter, stay in, exit or re-enter States along their migration journey in an authorized manner) for cross-border forcibly displaced people [29]. Consequently many forcibly displaced children undertake long, difficult, and dangerous journeys using irregular pathways, often at the hands of exploitative and abusive smugglers.	Irregular migration pathways by land and sea may increase a child's risk of disease, injuries, malnutrition, psychological trauma, and death. Migrants who arrive in countries by irregular routes may experience limitations on their rights and entitlements which may adversely affect their health and wellbeing.	Countries should increase and enhance pathways for humanitarian migration. The International Office for Migration recommends: enhanced flexibility in immigration procedures for humanitarian reasons; development of humanitarian admission and protection programmes; provision of proper documentation and legal identity services; establishment of special residence categories for forcibly displaced people; and increased clarity and broadened scope of formal regulations [29].
Border security	Pushbacks of migrants seeking sanctuary are occurring at European country borders [[23], [24], [25]]. In addition, criminalisation of humanitarian assistance to migrants at sea is a growing trend [30]. These policies violate the principle of non-refoulement under international human rights law.	Forced return to the dangers migrants were attempting to flee increases their risk of physical and mental trauma and death [24,25] Obstruction of sea rescue missions increases a child's risk of dehydration, hypothermia, injuries, and death from drowning.	Countries should ensure good faith implementation of their human rights obligations. Strengthening of independent border monitoring mechanisms can increase transparency and accountability of migration control policies and practices [31]. Governments should refrain from passing legislation that allow pushbacks or criminalise search and rescue at sea, and repeal existing legislation that contravenes human rights obligations [31].
Asylum process	Asylum policies are restrictive and complex with some countries systematically breaching international and EU standards for migrants' rights [22]. Asylum interviews are rarely trauma-informed and often not adapted to the child's age, capacities, or maturity [32]. It can be difficult for people seeking asylum to provide the evidence required to support their claims and there is often a long wait for a decision on asylum claims. Children seeking asylum may be subject to age assessments in cases where immigration officials doubt their given age or when there are no documents to prove their birth date. Quality and methods of assessment vary across Europe. There is no 100 % accurate or reliable method for determining age and there are concerns over the medical, legal, and ethical acceptability of some methods (e.g. x-rays) [33].	The asylum processes, age disputes and the associated instability and uncertainty adversely effects the mental health of child migrants, particularly unaccompanied minors [34]. Asylum interviews risk retraumatising children who have experienced trauma and loss. Medical methods involving ionising radiation can expose children to avoidable health harms, including increased future cancer risk. Consequences of incorrectly identifying a child as an adult can lead to safeguarding risks and may impact the child's right to access health and social care where entitlements differ by age.	Children should receive age, language and literacy appropriate information, advice and legal representation on all aspects of the asylum process. Asylum interviews should account for a child's age, gender, cultural background, maturity, psychological development, circumstances of flight and mode of arrival [35]. Immigration officers should be trained in trauma-informed and child friendly interview approaches. Where needed, trained interpreters should be present and non- verbal communication methods should be used [35]. Strengthening institutional and human capacity across the asylum claim process could reduce the time taken to issue a decision on a claim. The Council of Europe has produced guidance on a human rights based approach to age assessment [36].
Child immigration detention	Despite international law to the contrary, at least 40 countries within the WHO European Region detain children for immigration purposes [37].Conditions in many immigration detention facilities do not meet internationally recommended standards. Concerns include: inappropriate prison-like environments; poor sanitation and hygiene; lack of adequate food; safety concerns; restricted access to outdoor space, educational and leisure activities, and communication channels; limited healthcare; and exposure to violence and racism [38]. Efforts by European countries to restrict migration to the Region results in children being held in detention centres outside Europe (e.g. Libya) [39].	Child immigration detention has a detrimental impact on children's mental health; depression, anxiety, post traumatic stress disorder (PTSD), sleep disorders, somatic complaints, self-harm and suicidal ideation are reported consequences [38,40]. Detention has also been linked to developmental delay and regression, and emotional and behavioural problems [38,40]. Poor physical health has been linked to environmental conditions, insufficient nutrition, and inadequate medical care [38].	Legal frameworks should be adopted to prohibit the detention of children based on their or their families' migratory status. Countries should adopt rights- and community-based alternatives to detention, which involve positive engagement with migrant children in a person-centred approach to case management [38]. The International Detention Coalition advocates countries use the Child Community Assessment and Placement model [41]. Alternatives to detention have been shown to be more humane, more effective in protecting and improving health and wellbeing, more cost effective, and result in higher rates of case resolution [38].
Healthcare access	Healthcare entitlements are often restricted and usually dependent on immigration status [42]. Realisation of entitlements is impeded by: misapplication of complex health policies; administrative and access barriers; wrongful discrimination by healthcare gatekeepers; lack of human resources in primary healthcare; and the deterrent to access services created by distrust, the fear of unaffordable out-of-pocket payments and data sharing for immigration control [7,43].	Restricted access to healthcare increases the risk of morbidity and mortality. Access barriers experienced by pregnant migrant women results in higher risk of adverse perinatal outcomes [44]. Children miss out on vaccination and screening programmes [45,46]. Delayed presentation for injury or illness is associated with worse health outcomes. Parental access to healthcare is important to ensure their ability to care for their child and promote their wellbeing.	In keeping with the UN Sustainable Development Goals, countries should extend the legal entitlements to universal health coverage to all of their resident population, irrespective of immigration status. Inclusive non-emergency healthcare provision can be cost-saving, and reduce the risk of disease progression to more expensive-to-treat conditions requiring specialist and in-patient care [47]. Healthcare providers should deliver on healthcare entitlements and ensure they provide inclusive quality child-friendly services free of racism, discrimination and xenophobia [19].
Education	Despite access to basic education being a child's right, the type, quality and duration of schooling offered to child migrants depends on their legal status rather than their educational needs [48]. Concerns include: delays in providing education; substandard education provision in reception centres; administrative challenges to school enrolment; and the deterring practice of data sharing by schools for immigration control [48]. Some European countries explicitly limit or exclude undocumented children's right to schooling [48]. Lack of language, psychosocial and cultural mediation support, institutional and interpersonal racism and discrimination, and socioeconomic inequalities contribute to disengagement with education and higher rates of early school leaving amongst refugee and migrant children [48].	Health benefits of school attendance include: access to school based health services, screening and vaccination programmes; social interaction; promotion of cognitive, personal, social and emotional development; advancement of health literacy; nutritional support (where school offer includes school meals); reduced risk of exposure to abuse and exploitation; opportunity for professionals to recognise a child in need; and acquisition of skills and qualifications which increase employability and financial security associated with improved long-term health outcomes.	All children should have a legal right to education in their host country irrespective of immigration status, and receive the support required to access this right. European country examples of positive policy and practice include: mitigating financial disadvantages to reduce inequalities in education access and quality; multi-stakeholder coordination to support school enrolment; additional language support; strengthening of education provider's capacity to facilitate integration; programmes to engage parents; mentoring schemes; tailored curriculums for newly arrived migrant children; and school partnerships with social services, municipalities and business sector to prevent early school leaving [48].
Family unity	In many European countries the definition of ‘family’ for reunification purposes is limited to parents and minor children [49]. This fails to account for the reality for many separated children whose parents may be dead, missing or imprisoned, and neglects the important role extended family plays in some cultures. Family reunification processes are often slow and complex and require documents difficult for forcibly displaced people to provide [49]. Some families face financial barriers to reunification [49]. Unaccompanied minors who apply for asylum may lose their right to family reunification if they turn 18 during the asylum process. Undocumented migrant children are often excluded from family reunification policies and in some countries, e.g. the UK, even children with protection status are not eligible to sponsor family members [50]. Failure to facilitate family reunification violates a child's right to family unity.	Presence of a caring family is protective of child health, development, and wellbeing [51]. Unaccompanied migrant children are at increased risk of violence, abuse, neglect, trafficking and exploitation and the associated health harms [15]. Absence of family increases a child migrant's risk of experiencing psychosocial and mental health problems [15]. Population benefits of family unity include social and economic cohesion, which can have positive health impacts [52].	Countries should uphold a child's right to family life through migration policy and practice that prevents separation of children from families and facilitates family reunification. Recommendations include: a broader definition of family; reasonable requirements of potential sponsors and applicants; access to age, gender and culturally appropriate information; free legal counselling and assistance; minimal waiting times to process applications; removal of financial barriers; and improved international cooperation [52]. The Council of Europe has compiled examples of positive policy practice around family reunification for refugee and migrant children [53].
Poverty and destitution	Immigration policies that restrict access to the legal labour market, social security, public services, education, and training increase the risk of child migrants experiencing poverty [54,55]. Within the EU poverty is estimated to be more than 200 % higher amongst migrants than the host population [55]. Immigration policy designed to push people into poverty and destitution as a deterrent to others planning to travel to the country is not only ineffective but also undermines child rights [55].	Poverty adversely affects child wellbeing and development, including their cognitive ability, school achievement, mental health, and social-behavioural development [56]. Poverty indirectly impacts child health through its effect on parental mental health, parenting capacity, housing and the physical home environment [56].	Reduction in time spent subject to immigration control, access to social security and public services, policies that increase income redistribution, family reunion, parental/care-giver right to work, quality housing support, and education and training opportunities can protect child migrants against poverty and destitution [54,55].
Child labour	Child labour as one of the critical child protection risks related to forcibly displaced and stateless children [57]. Separated, unaccompanied and undocumented migrant children are at particular risk, as are child migrants of certain national and ethnic origins as a consequence of racism and discrimination [58]. Policies that expose child migrants to poverty and destitution; restrict their access to education, regulated employment and public services; or exclude them from child protection systems will increase their risk of exploitative child labour [59]. Non-permanent resident status and lack of identity documents often exclude migrant children from government work protections. Hostile policies that generate fear of detention or deportation prevent children reporting exploitation or seeking help [58]. A 2023 report on the state of global child labour presents country case examples highlighting the extent of child migrant labour in Europe [60].	By definition child labour is ‘work that deprives children of their childhood, potential and dignity, and that is harmful to physical and mental development’ [61]. Specific health impacts will depend on the type of labour and environmental conditions the child is subjected to. The often informal nature of the labour exposes children to poor working conditions, long hours, low pay, delayed wages, and dangerous environments. Risks associated with hazardous child labour include death, illness, injury, disability, and psychological damage [62].	In compliance with UNCRC and International Labour Organization Conventions countries should implement legislative, enforcement, administrative, social and educational measures to protect all children from child labour harms. Recommendations include: legislation that meets international standards on permitted minimum working age and prohibition against forced labour, child trafficking, commercial sexual exploitation and use of children for illicit activities; enforcement of laws and regulations on child labour through competent inspectorates and imposed penalties for violation; monitoring and publication of child labour and enforcement data; policies which address poverty as a driver of child labour; strengthening and expansion of child protection systems to include migrant children; access to education, technical, vocational and life skills training irrespective of immigration status; and employment opportunities in the formal sector [60,63].
Accommodation/housing	Living standards and accommodation entitlements across the Region are variable. Some children are living in slums and refugee camps where they are experiencing weather extremes, hunger, lack of education, and exposure to violence, abuse and exploitation. Many Member States provide transit centres, accommodation centres and private housing for children and families seeking asylum but these are often below the required standard [[64], [65], [66], [67]]. Many forcibly displaced people in Europe have to pay for private accommodation, for example almost all international protection applicants in Türkiye, which increases the risk of destitution and homelessness amongst forcibly displaced children [67]. Of particular concern is the widespread lack of adequate accommodation and systematic care provided to unaccompanied minors [68].	Overcrowding increases the risk of communicable disease and heightened tensions which can lead to a volatile and potentially violent environment; lack of separate safe spaces and privacy increases the risk of sexual and gender based violence; mould, asbestos, poor sanitation and hygiene facilities and vermin increase the risk of diseases; unsuitable environments increases injury risks; and lack of access to cooking facilities increases the risk of malnutrition [65]. Housing insecurity and poor quality housing negatively impacts child development and mental health [69]. Frequent relocation hinders integration, continuity of care, and education access.	To uphold the right of every child to a ‘standard of living adequate to the child's physical, mental, spiritual, moral and social development’ countries should strengthen the entitlements foreseen in national legislation and ensure their proper implementation [5]. UNICEF calls on Member States to: ensure that housing standards for refugee and migrant children meet the health and safety requirements set by existing international and national standards; develop strategies aimed at guaranteeing access to private housing and flats for families and children; implement safeguarding rules and ensure staff receive adequate training, monitoring and support; invest in extending existing social services for children, youth and families; and provide family-based housing solutions for unaccompanied and separated children [64].

## 6 Upholding the right to facilities for the treatment of illness and rehabilitation of health

### 6.1 Overcoming health system challenges to addressing needs

Underfunded public health systems, limited specialist health services and Region wide health and care workforce challenges are barriers to addressing the health needs of displaced children. While the majority of European countries' have adequate resource to meet the needs of children seeking sanctuary there, migrants entering the Region via the Eastern Mediterranean, Western Balkans and Eastern borders route will often travel through or settle in countries with some of the lowest GDP per capita in the WHO European Region. Health spending in countries in Central, Eastern and Southern Europe can be five times lower than in high-income countries in Western and Northern Europe [70]. Türkiye hosts the highest numbers of child refugees; health spending is EU 1000 per capita and it has the Region's lowest health professional density at 54.3 per 10,000 population [70,71]. For comparison Germany, who receives 40.6 % of EU first time child asylum applicants, is better resourced to provide care with health spending at EU 5317 per capita and over 175 health professionals per 10,000 population [8,70,71]. Health systems across the Region are still recovering from the Covid-19 pandemic but emerging geopolitical and economic conditions contribute to government deprioritisation of health at a time when health crisis preparedness (including response to conflict and disaster displacement) should be a top concern. Investment in health system strengthening and resilience, primary care and child health services, alongside enhanced workforce planning, training and regulation for workforce growth and development is needed to improve the quality of care for all children - in both countries' host and migrant populations.

Healthcare entitlement restrictions are not the answer to capacity challenges. Not only do they violate the rights of displaced children, they fail to recognise that inclusive non-emergency healthcare provision to refugees, asylum-seekers, and undocumented migrants can generate savings in both medical and non-medical expenses [19,47]. Accessible healthcare reduces the risk of ill health progressing to more complex conditions that require costly specialist treatment and increase pressure on emergency departments, inpatient services and social care. Integration of skilled forcibly displaced migrants into local health workforces could help address the limitations in human resources for health and improve timely delivery of services. In 2022 the European Commission issued a recommendation on the recognition of qualifications for people fleeing Russia's invasion of Ukraine, encouraging its Member States to explore how people enjoying temporary protection can, where appropriate, be employed in healthcare services [72]. UN Agencies have supported countries to realise this recommendation [73].

Where national entitlements and recommendations for inclusive services exist, it is essential they are realised in practice. For example, General Practice surgeries in the UK have wrongly refused to register migrants unable to provide identity and proof of address documents, and the country's complex NHS Overseas Visitor Charging policy has led to racial profiling and child migrants experiencing delayed treatment and refusal of care due to eligibility mistakes [74,75]. The perpetuation of racism, xenophobia and discrimination within European healthcare systems and the failure to address language, cultural, social, financial, and administrative barriers to care must be urgently rectified to ensure the health system is accessible and acceptable to child migrants [19]. Child migrants and their families need information and support to navigate unfamiliar health systems.

Some countries provide healthcare to displaced children in a system external to their national health system [76]. While there are benefits to specialist services tailored to the needs of displaced children and their families, parallel health systems can drive inequity between population groups. Most often displaced children will receive poorer quality services than the general child population, for example through inadequate healthcare provision in refugee camps or immigration detention centres. Countries should endeavour to provide migrant-sensitive healthcare which is integrated into the mainstream health-system, grounded in policy, with commitment from governments, public agencies, health-system leaders, health workers and NGO providers. Education, guidance and referral pathways to support holistic assessment and management of the health and wellbeing needs are needed. Technical guidance and standards exist and should be adapted and applied [[77], [78], [79], [80]]. Health professionals should be trained in trauma-informed care, diversity responsiveness, intercultural competencies, implicit bias, safeguarding and child protection pathways. Interpreters and translated health materials should be provided where needed to reduce the health harms associated with language barriers [81].

A crucial step to building a health-system responsive to the needs of children who have been forcibly displaced is to address the current data gap for this population. Countries need to collect quality health data disaggregated by migration status, age and sex to inform responsive health policy and practice. The health data of displaced children should be integrated into the host nation's health information systems and countries across the European Region should implement data sharing agreements to facilitate continuity of care for children on the move.

Country willingness to address the health needs of displaced children varies across the WHO European Region. The level of healthcare coverage entitlements, service accessibility and responsiveness differs between countries but all have a way to go if they are to achieve delivery of migrant-sensitive healthcare that is equitable to the quality of that received by the host population [42].

### 6.2 Assessing the health and wellbeing needs of children who have been forcibly displaced

All children should have a health assessment on arrival in a host country to identify and address their needs. Documented health needs of displaced children include: communicable diseases; incomplete immunisation histories; undiagnosed and poorly managed non-communicable diseases; malnutrition and micronutrient deficiencies; anaemia as a consequence of malnutrition, parasitic infections or haemoglobinopathy; musculoskeletal complaints; physical trauma; sexually transmitted infections; adolescent pregnancy; oral disease; problems associated with female genital mutilation (practised in some African and Middle Eastern countries); post-traumatic stress disorder; anxiety; depression; internalising and externalising behaviours; and sleep disorders [7,15]. Health assessments should also screen for development and disability needs. Health, nutrition, personal environment, and exposure to traumatic events are important determinants of child development therefore adversities associated with forced displacement may interfere with critical stages of a child's social, emotional, physical and intellectual development [82,83]. Disabled forced migrants are largely invisible from the data and have a history of being neglected in policy and service response, despite rehabilitation for disabilities being described as amongst the most pressing needs experienced by child migrants [80,84]. Being disabled and a migrant can result in dual disadvantage for a child through loss of a family's social support networks; cultural and linguistic differences impacting recognition of needs; access barriers to formal services; deprivation; and increased stigma, marginalisation, and discrimination [85,86]. Displaced children with neurodevelopmental disabilities are especially vulnerable to challenges associated with accessing healthcare and education [87].

A ‘think-family’ approach should be applied to health assessments of newly arrived accompanied children in recognition that child and parent/caregiver health is inter-related [88]. Screening and health promotion should be inclusive of caregivers as good health improves parenting capacity, thus enhancing child wellbeing [88]. Refugee and asylum-seeking parents have an increased vulnerability to mental disorders because of the traumas they have been exposed to and the challenges associated with social integration in the host country [89,90]. Parental mental disorders may impair development of positive parent-child relationships and secure parent–child attachment, impact parental ability to recognise and respond to their child's needs, and increase the risk of the child developing mental health problems [89,91]. Therefore, addressing parental needs is a necessary step to meeting those of the child. Additionally, there should be urgent assessment and management of displaced pregnant women who often experience maternal multimorbidity and higher rates of newborn mortality and morbidity as a consequence of physical, psychological and social conditions and healthcare barriers [92]. It has been proposed that poorer maternal healthcare for migrant women could be contributing to the plateauing/rising infant mortality rate in some countries in the Region [93]. Education and support for newly arrived migrant families on the host country's parenting norms and safeguarding laws in the context of cultural differences, and the health and social support available to them could promote positive parenting and empower families to thrive in their new environment.

Unaccompanied and separated children have additional needs to children who have migrated with caregivers [15]. Unaccompanied children are at a higher risk of mental health issues such as PTSD, depression, anxiety, self-harm and suicide due to experiences of violence, trauma, loss of family, uncertainty about the future, and a lack of emotional support from families which could buffer against the effects of stress [15,94]. Unaccompanied and separated children are at particular risk of human trafficking for labour and sexual exploitation and assessments should include evaluation for risk factors for and signs of exploitation and abuse [15].

## 7 Limitations

There are limitations in the scope of this paper and the authors wish to highlight the need for further research, policy evaluation and creation of an evidence-base of good practice not only for children in Europe who have been forcibly displaced across international borders but the wider population of children impacted by migration as discussed in Panel 2.

**Table:** 

Panel 2: Limitations in the scope of this paper
While outside the scope of this paper the high number of internally displaced children within Europe is also of concern. More than 2.5 million children have been internally displaced within Ukraine since the 2022 Russian Federation invasion [95]. In Türkiye 660,000 children were internally displaced following a series of earthquakes in 2023 and an estimated 205,000 were yet to return home a year later [96]. Internally displaced children have often experienced physical and psychological trauma and have associated health needs. Their country's health, social care, and education systems may have been adversely impacted by the conflict or disaster that initiated the displacement. This, alongside increased service demands, may limit a country's ability to adequately respond to the needs of internally displaced children. While this paper focuses on children who have been displaced across international borders and face additional challenges associated with restricted rights and entitlements, language barriers, and discrimination, it is important to acknowledge internally displaced children as a population group who must not be forgotten in European research, policy and planning activities.
Children who have been forcibly displaced and returned home are another group with high needs but excluded from this paper. Some children may be returned against their will to face the dangers they were fleeing following failed asylum claims. For others a return home may be welcomed but family reunification and reintegration may present challenges, and countries whose infrastructure and environments have been damaged by events that precipitated displacement may experience difficulties meeting the health and wellbeing needs of returning children.
It is important to acknowledge that Member States are contributing to the forced displacement of children outside the WHO European Region. For example, Israel's military action in the Occupied Palestinian Territory, and European Member States' global arms trade are contributing to forced displacement and child rights violations outside the Region. Likewise, failure to take meaningful action to address climate change concerns will result in an escalation of climate related displacement. European countries should endeavour not only to protect all children within its borders but also to cease harm to children outside of them.
Child migrants in Europe, irrespective of reason for migration, often experience racism, xenophobia and discrimination at structural, institutional and interpersonal levels and frequently experience social and health inequalities [19]. Urgent action is needed to address inequities.
Also outside the scope of this paper is consideration of the health and wellbeing of children in Europe who are “left behind” by one or both parents migrating for work, study, or to seek a better life. In Kyrgyzstan this is the situation for 10 per cent of children [97]. A country's labour and migration policies can influence whether parental migration can have positive or negative impacts on a child's development, economic status, opportunities, and wellbeing [97]. WHO European Member States need to ensure this cohort of children are not excluded from policy decision-making.

## 8 Next steps

The disregard of child migrants' rights played out in European country politics demands a reaction. States must be held accountable to agreed international human rights using legal and political instruments, but also by the power of civil society including health professionals and academics. Civil society organizations (CSOs), particularly those involved in rescue operations and humanitarian aid, play a crucial role in supporting migrants and refugees. Instead of criminalising these organizations, states should recognise their contributions and provide structural and financial support to enable their work. Independent monitoring mechanisms proposed by countries to address current failings can only be effective if adequately resourced, truly independent and if operating in an environment where there is genuine political will to uphold migrant and refugee rights [98]. There is precedent that things can be done differently. The Russian Federation's invasion of Ukraine triggered the EU to activate the Temporary Protection Directive, providing immediate access to healthcare to displaced Ukrainian citizens. Many countries mobilised resources, produced guidance and translated health information to meet the health needs of displaced Ukrainians and enable continuity of care and sustained access to medications [99]. This positive response demonstrates that when there is political will, Europe is able to take coordinated action to welcome and support displaced migrants with fairness and humanity.

The Region needs to develop a response to displacement that is grounded in solidarity and shared responsibility, one that alleviates the strain on countries receiving high numbers of migrants and upholds the rights of people seeking sanctuary. Countries should not be outsourcing their responsibility to protect refugees, a criticism of the controversial 2016 Türkiye-EU deal. Instead, cross-country collaboration is required to address the drivers of forced migration; increase availability of safe and legal routes for refugees; and ensure health systems across the Region have the data, resource and capacity required to respond to the needs of displaced children. Support is needed from UN agencies for migration preparedness, rapid assessment and response for countries experiencing high numbers of newly arrived migrants, and long-term planning for integration.

Countries should promote and protect the rights of all children, irrespective of migration status, and this should be evident in national policy and practice. Table 2 presents recommendations on how this could be achieved. Essential policies supporting a child's right to health include: provision of child and family-centred community alternatives to refugee camps and immigration detention; provision of healthcare and education entitlements equitable to children of the host nation; and protection of children from violence and exploitation. Action must be taken to combat racism, xenophobia and discrimination towards child migrants at structural, institutional and interpersonal levels. Countries need to address data gaps by establishing and integrating data collection into existing systems in order to develop a multi-sector system-wide response with migration as a principal consideration in the design of service delivery, policy and research. To ensure displaced children have access to the treatment and rehabilitation needed to achieve good health, countries should deliver responsive quality and inclusive trauma-informed healthcare that accounts for language needs, cultural diversity and safeguarding risks. There needs to be investment in human resources to enable quality healthcare delivery and health professionals should be equipped with the necessary knowledge, tools and resource.

Promoting and protecting the health of children who have been forcibly displaced is not only morally, and often legally, the right thing to do, but it also provides an opportunity for national economic growth and productivity. Investment in all children and young people across the Region is an investment in Europe's future.

## Funding

This paper did not receive any specific grant from funding agencies in the public, commercial, or not-for-profit sectors.

## Declaration of competing interest

The authors declare that they have no known competing financial interests or personal relationships that could have appeared to influence the work reported in this paper.
